# p27kip1 expression and phosphorylation dictate Palbociclib sensitivity in *KRAS*-mutated colorectal cancer

**DOI:** 10.1038/s41419-021-04241-2

**Published:** 2021-10-15

**Authors:** Gian Luca Rampioni Vinciguerra, Alessandra Dall’Acqua, Ilenia Segatto, Maria Chiara Mattevi, Francesca Russo, Andrea Favero, Roberto Cirombella, Giorgia Mungo, Davide Viotto, Javad Karimbayli, Margherita Pesce, Andrea Vecchione, Barbara Belletti, Gustavo Baldassarre

**Affiliations:** 1 Division of Molecular Oncology, Centro di Riferimento Oncologico di Aviano (CRO), IRCCS, National Cancer Institute, Aviano, Italy; 2 Faculty of Medicine and Psychology, Department of Clinical and Molecular Medicine, University of Rome “Sapienza”, Santo Andrea Hospital, Rome, Italy

**Keywords:** Colorectal cancer, Cell growth, Cell signalling

## Abstract

In colorectal cancer, mutation of *KRAS* (RAS^MUT^) reduces therapeutic options, negatively affecting prognosis of the patients. In this setting, administration of CDK4/6-inhibitors, alone or in combination with other drugs, is being tested as promising therapeutic strategy. Identifying sensitive patients and overcoming intrinsic and acquired resistance to CDK4/6 inhibition represent still open challenges, to obtain better clinical responses. Here, we investigated the role of the CDK inhibitor p27^kip1^ in the response to the selective CDK4/6-inhibitor Palbociclib, in colorectal cancer. Our results show that p27^kip1^ expression inversely correlated with Palbociclib response, both in vitro and in vivo. Generating a model of Palbociclib-resistant RAS^MUT^ colorectal cancer cells, we observed an increased expression of p27^kip1^, cyclin D, CDK4 and CDK6, coupled with an increased association between p27^kip1^ and CDK4. Furthermore, Palbociclib-resistant cells showed increased Src-mediated phosphorylation of p27^kip1^ on tyrosine residues and low doses of Src inhibitors re-sensitized resistant cells to Palbociclib. Since p27^kip1^ showed variable expression in RAS^MUT^ colorectal cancer samples, our study supports the possibility that p27^kip1^ could serve as biomarker to stratify patients who might benefit from CDK4/6 inhibition, alone or in combination with Src inhibitors.

## Introduction

Colorectal cancer (CRC) represents the third most common cancer and the second leading cause of cancer-related death worldwide [1, 2]. About 20% of patients have synchronous metastases at diagnosis and about 35% of patients develop metastases after a curative intent treatment. Many different features, regarding clinical aspects, tumor characteristics and molecular profile, may contribute to CRC heterogeneity and, eventually, correlate with treatment response and patients’ prognosis [1–4]. Approximately 50% of CRC patients harbor *RAS* mutations (RAS^MUT^), which determines a constitutive activation of Ras/Raf/MEK/ERK signaling pathway. RAS^MUT^ CRC are intrinsically resistant to anti-epidermal growth factor receptor (EGFR] targeted therapy [1, 2]. Similarly, efforts made to develop treatments for RAS^MUT^ tumors, targeting different effectors of Ras/Raf/MEK/ERK pathway obtained limited success [5–7]. Increased signaling through upstream receptor tyrosine kinases or activation of parallel signal transduction cascades that reactivate ERK have been proposed as resistance mechanisms [8, 9] that invariably lead to cell cycle progression, *via* increased cyclin D and decreased p27 expression [10, 11].

Chemotherapy, with or without the addition of Bevacizumab, remains the standard treatment for RAS^MUT^ CRC patients, who currently have very few therapeutic options. Therefore, identification of new clinically relevant targets and biomarkers represent an urgent need for these patients [1–4].

Recent studies have tested the antitumor activity of the combination of anti-cell cycle drugs and inhibition of the Ras pathway, in RAS^MUT^ tumors [12–15]. In this attempt, small inhibitor molecules blocking the activity of cyclin dependent kinases (CDK) 4 and 6 have been used. Among them, Palbociclib is a selective CDK4/6 inhibitor (CDK4/6i) already approved for the treatment of metastatic hormone receptor positive breast cancers [16]. CDK4/6i have displayed encouraging results in preclinical models of RAS^MUT^-driven tumors [12, 13, 17–19]. However, initial efficacy was often accompanied by rapid onset of acquired resistance, and, to date, no molecular biomarker has been validated to identify patients who might respond to CDK4/6i treatment.

p27^Kip1^ (hereafter p27) is a cell cycle inhibitor, member of the CIP/KIP family of CDK inhibitors. p27 is an haploinsufficient oncosuppressor and its functions can be both CDK-dependent and -independent [20, 21]. In human cancers inactivation of *CDKN1B* gene, encoding for p27, is rarely due to mutation or deletion [22, 23]. Conversely, reduction and/or mislocalization of p27 protein are very frequent and correlate with tumor aggressiveness and poor prognosis [20, 21]. p27 protein has a dual role in regulating cyclin D-CDK4/6 activity. It acts on one side as a potent inhibitor for these complexes and on the other p27 is required for proper complex assembly and stabilization [24–27]. The most accepted explanation is that post-translational modification of p27 proteins might dictate its inhibitor/non-inhibitor role. Indeed, p27 is a direct target of many intracellular pathways operating downstream of Ras (*e.g*. MAPK, PI3K) that lead to p27 protein phosphorylation and subsequent degradation or cytoplasmic delocalization [28]. Also non-receptor tyrosine kinases may directly phosphorylate p27 on tyrosine, thereby altering its affinity and ability to bind and inhibit cyclin-CDK complexes [26, 29–31]. Among others, BCR-ABL, Brk and Src family members phosphorylate p27 on tyrosine 74 (Y74), 88 (Y88) and 89 (Y89), early after mitogen stimulation [26, 29, 30]. Very recent structural biology data have demonstrated that pYp27/CycD/CDK4 complexes are refractory to inhibition by CDK4/6i and, by retaining p27, indirectly unleash CDK2 from p27 inhibition [32].

A complex regulatory loop exists between Ras and p27 to control cell cycle progression in response to mitogenic stimuli [28, 33–35]. Recent evidences in RAS^MUT^ lung tumor cells demonstrated that prolonged exposure to Palbociclib induced an increased expression of p27 that was then able to form an active complex with cyclin D1 and CDK6, contributing to the resistant phenotype [14].

Whether these mechanisms may be working also in vivo and, particularly, in RAS^MUT^ CRC, is still unexplored. Here, we test this possibility and investigate the role of p27 in RAS^MUT^ CRC response to Palbociclib.

## Materials and methods

### Human specimen collection, study approval and histological analysis

Formalin-fixed and paraffin-embedded (FFPE) specimens (*n* = 201) were retrospectively collected from patients with primary and metastatic colorectal cancer upon signing a written informed consent, in accordance with recognized ethical guidelines and followed by approval of University of Rome “Sapienza” Santo Andrea Hospital (Rome, Italy).

For immunohistochemical analysis, sections from FFPE specimens were stained with anti-p27, Src and pSrc^Y416^ antibodies as better specified in supplementary methods. Immunofluorescence analyses were performed as previously described [36] and as reported in Supplementary Information.

### Cell culture and transfection

All colorectal cancer cell lines were maintained in RPMI 1640 (Sigma-Aldrich) supplemented with 10% fetal bovine serum (FBS, Carlo Erba) and 1% penicillin and streptomycin (PS, Lonza). All cell lines have been authenticated using the Cell ID TM System (Promega). SW480 Palbociclib-resistant cells were generated by continuous treatment with increasing doses (from 0.25 to 2 µM) of Palbociclib (PD-0332991, Clinisciences) [14]. Kill curve analyses with Palbociclib and Cetuximab (Erbitux®, from Merck) were performed as previously described [37, 38]. Normal and transformed WT or p27^KO^ 3T3 fibroblasts and 293FT were cultured in DMEM (Sigma-Aldrich) supplemented with 10% FBS (Carlo Erba) and 1% PS (Lonza).

### qRT-PCR, Western blot, immunoprecipitation and kinase assay

RNA extraction, qRT-PCR, protein lysates, immunoprecipitation (IP), Western blot analyses and in vitro kinase assays were performed as already described [36, 39, 40]. Detailed methods are in Supplementary Information.

### Animal experimentation

Animal experimentation was reviewed and approved by the Centro di Riferimento Oncologico di Aviano (CRO) Institutional Animal Care and Use Committee (OPBA). All animal experiments were conducted in adherence to international and institutional committee ethical guidelines. NOD.CB17-PrkdcSCID (NSG) male mice (Charles River Laboratories) were orthotopically injected with HCT-116-Luc2 (Caliper) cells and treated with Palbo and Saracatinib, alone or in combination, as described in Supplementary Information. Combined macroscopic and pathological analyses of the mouse organs were performed as described in [41].

### Statistical analyses

Statistical significance, averages, median, and SD were determined by using GraphPad PRISM software (version 6.01), using the most appropriate test, as specified in each figure. Significance is indicated by a *p* < 0.05. Detailed methods can be found in Supplementary Information.

## Results

### Expression of p27 does not correlate with mutation of *RAS* in colorectal cancer

**Fig. 1:**
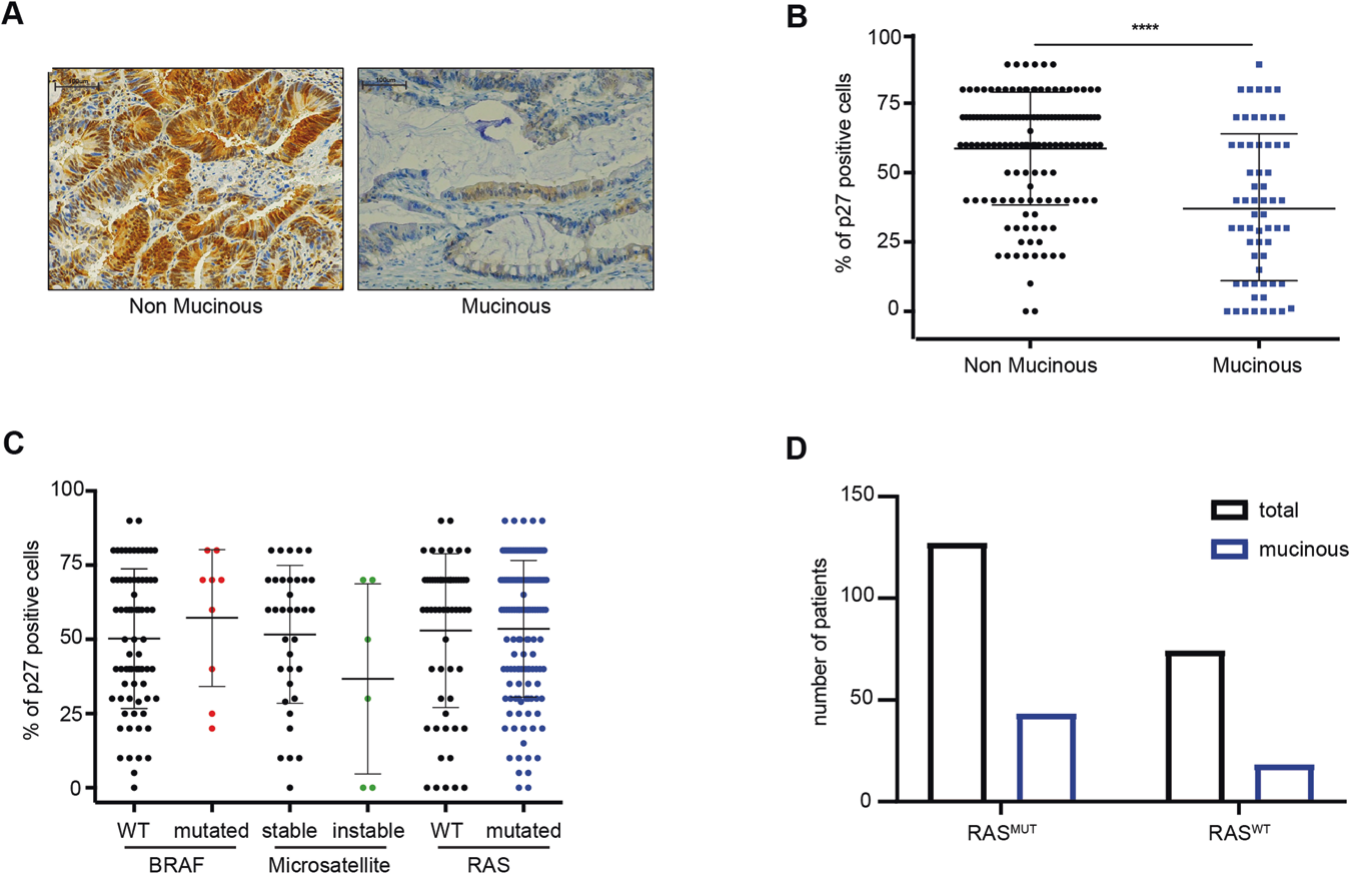
Expression of p27 is independent from most clinical and molecular parameters in colorectal cancer (CRC). **A** Immunohistochemistry (IHC) analysis of p27 expression in *non* mucinous (left) and mucinous (right) CRC (20x magnification). **B** Percentage of p27 positive cells in *non* mucinous and mucinous CRC samples. In **B** and **C,** each dot corresponds to a CRC sample. Mann–Whitney test was used for statistical analysis (****p < 0.0001). **C** Percentage of p27 positive tumor cells classifying CRC samples with different molecular features, as indicated. **D** Graph reports the number of all analyzed tumors (black) and tumors with mucinous features (blue), with respect to their *RAS* mutational status.

The gene encoding for p27, *CDKN1B*, is rarely mutated in cancer and its regulation mainly occurs at post-transcriptional level [22, 23]. We thus assessed p27 expression in a large internal collection of CRC specimens (Table 1) by immunohistochemistry (IHC) analyses and observed a significant association between low p27 expression and CRC with mucinous features that display a very aggressive clinical behavior (Fig. 1A and B). On the other hand, p27 expression levels did not specifically associate with microsatellite-instability, *BRAF* (BRAF^MUT^) or *RAS* mutation (RAS^MUT^, including 114 *KRAS* and 7 *NRAS* mutated tumors) (Fig. 1C). CRC with mucinous features were evenly distributed between RAS^MUT^ and RAS^WT^ tumors (Fig. 1D). Altogether, these analyses indicated that p27 expression is highly variable in CRC and its low levels associate with clinical aggressive behavior but not with specific molecular alterations.

**Table 1:** Clinical and pathological features of CRC samples. Table describes the distribution of human colorectal tumors collected at University of Rome "Sapienza", according to high/low levels of p27 respect to the expression average (52% of neoplastic cells) and to the indicated clinico-pathological features. p-value was calculated using Fisher’s exact test.

Clinic-pathological features of CRC tumors		Low p27	High p27	*p-*value
		(*n* = 86)	(*n* = 115)	
Age	Average (range)	65 (23–86)	67 (32–87)	0.419
Sex	Female	36 (42%)	40 (35%)	0.305
	Male	50 (58%)	75 (65%)	
Cancer Site	Primary	77 (90%)	94 (82%)	0.124
	Metastasis	9 (10%)	21 (18%)	
Grading	1–2	20 (23%)	33 (28%)	0.201
	3–4	56 (65%)	60 (52%)	
Stage	I–II	15 (17%)	10 (9%)	0.094
	III–IV	70 (81%)	96 (83%)	
RAS	Mutated	57 (66%)	64 (56%)	0.279
	Wild type	29 (34%)	45 (39%)	
BRAF	Mutated	3 (3%)	7 (6%)	0.219
	Wild type	38 (44%)	37 (32%)	
Microsatellite	Instable	4 (5%)	1 (<1%)	0.133
	Stable	15 (17%)	19 (17%)	
Histotype	Mucinous	42 (49%)	19 (17%)	0.000001
	Non-mucinous	44 (51%)	96 (83%)	
Tumor Edge	Pushing	5 (6%)	18 (16%)	0.941
	Infiltrative	7 (8%)	24 (21%)	
Neoadjuvant	No	74 (86%)	91 (79%)	0.987
	Yes	5 (6%)	9 (8%)

### Expression of p27 modulates sensitivity to CDK4/6 inhibitors, in vitro

Next, we tested p27 expression in a panel of 11 CRC cell lines. As observed in primary tumors (Fig. 1), p27 expression was variable among the different CRC cell lines, without clear association with the mutational status of *BRAF* or *RAS* (Supplementary Fig. S1A).

**Fig. 2:**
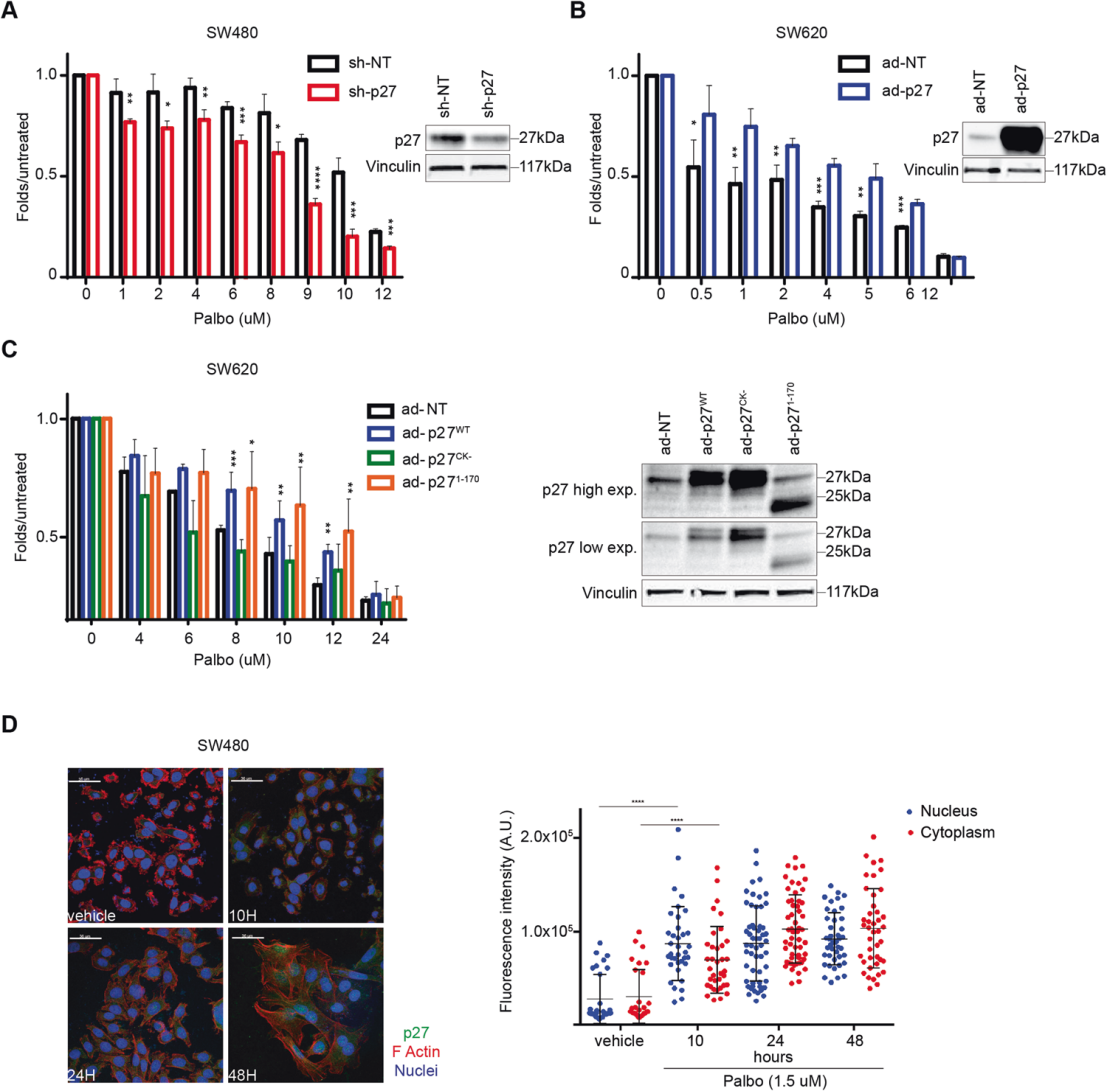
Expression of p27 modulates sensitivity to Palbo, in vitro. **A** Left: Dose–response curve of SW480 control (sh-NT) and p27-silenced cells (sh-p27), treated for 72 h with increasing doses of Palbociclib, as indicated. **B** Left: Dose–response curve of SW620 transduced with adenoviral particles expressing p27 (Ad-p27) or not (Ad-NT), treated for 48 h with increasing doses of Palbo. **C** Dose–response curve of SW620 cells transduced with control (Ad-NT), p27 WT (Ad-p27^WT^) and p27 mutants (Ad-p27^CK-^, Ad-p27^1-170^) expressing adenoviral particles, treated for 48 h with increasing doses of Palbo, as indicated. In **A**, **B** and **C** on the right are reported the Western Blot analyses evaluating p27 expression in the transduced as indicated cells and used in each dose-response curve. Vinculin was used as loading control. In all dose-response curves, cell viability was measured by MTS assay and data show the percentage of viable treated cells compared to untreated cells in 3 independent experiment. Student’s *t*-test was used for statistical analysis and asterisks indicate significant differences. *p < 0.05; **p < 0.01; ***p < 0.001; ****p < 0.0001. **D** Left: Immunofluorescence (IF) analysis of p27 (green), F-Actin (red) and nuclei (To-Pro-3, blue) performed on SW480 parental cells treated with vehicle or Palbociclib 1.5 μM for the indicated time points (hours, H). Right: quantification of p27 expression in nuclear and cytoplasmic compartment, performed using Volocity® (PerkinElmer) software. Each dot corresponds to one cell. Mann-Whitney test was used for statistical analysis (****p < 0.0001).

Interestingly, we observed that most high p27 expressing CRC cells were more resistant to Palbociclib (Palbo), compared to low p27 ones (Supplementary Fig. S1). This observation was particularly true among KRAS^MUT^ cells (Supplementary Fig. S1B). In this context p27 had a functional role, since p27 silencing in SW480 cells increased Palbo-sensitivity and p27 overexpression increased Palbo-resistance in SW620 cells (Fig. 2A and B). Intriguingly, altering p27 levels in RKO and HT-29 (KRAS^WT^/BRAF^MUT^) or Caco-2 (KRAS^WT^/BRAF^WT^) cells did not modify Palbo-sensitivity (Supplementary Fig. S1C–E), suggesting a specific role for p27 in RAS-mutated context. The use of 3T3 fibroblasts generated from p27^WT^ and p27^KO^ mice and transformed with different oncogenes, confirmed that KRAS^G12V^ transformed p27^WT^ cells were significantly more resistant to Palbo than corresponding p27^KO^ cells, while Palbo-sensitivity seemed quite independent from p27 when BRAF^MUT^ (V600E substitution) was overexpressed (Supplementary Fig. S2A–B). Interestingly, also transformation of 3T3 with v-Src elicited the same results of KRAS^G12V^, showing that p27^WT^ cells were more resistant to Palbo than corresponding p27^KO^ cells (Supplementary Fig. S2C).

p27 is a multifunctional protein with the N-terminus principally involved in the regulation of cyclin/CDK complexes and the C-terminus more involved in the regulation of protein stability, protein-protein interactions and microtubule and actin dynamics [20]. To understand which p27 domain could be responsible for Palbo response in CRC, we transduced SW620 cells (low p27) with p27 WT or mutant forms, unable to interact with cyclin/CDKs (p27^CK-^) or with stathmin (p27^1-170^), a cytosolic protein involved in the regulation of microtubule stability and vesicular trafficking and previously shown to interact with p27 (Fig. 2C) [20, 34, 35]. Overexpression of p27^CK-^ did not change cell sensitivity to Palbo, while the expression of p27^1-170^ acted as p27^WT^ and markedly increased Palbo-resistance, indicating that the binding of p27 to cyclin/CDK complexes was critical for the regulation of Palbo response, while the interaction with stathmin was not necessary in this context (Fig. 2C).

Palbo treatment induced an increase in p27 expression level, both in the nucleus and in the cytoplasm (Fig. 2D), preceded by a clear reduction in pRB^S780^ phosphorylation and accompanied by an increase also in CDK4 and CDK6 protein expression (Supplementary Fig. S3A). Likely, these differences were not due to changes in the transcription rate, since mRNA levels of p27, CDK4 and CDK6 did not significantly increased under Palbo treatment (Supplementary Fig. S3B).

### p27 modulates sensitivity to Palbociclib in an orthotopic model of CRC

**Fig. 3:**
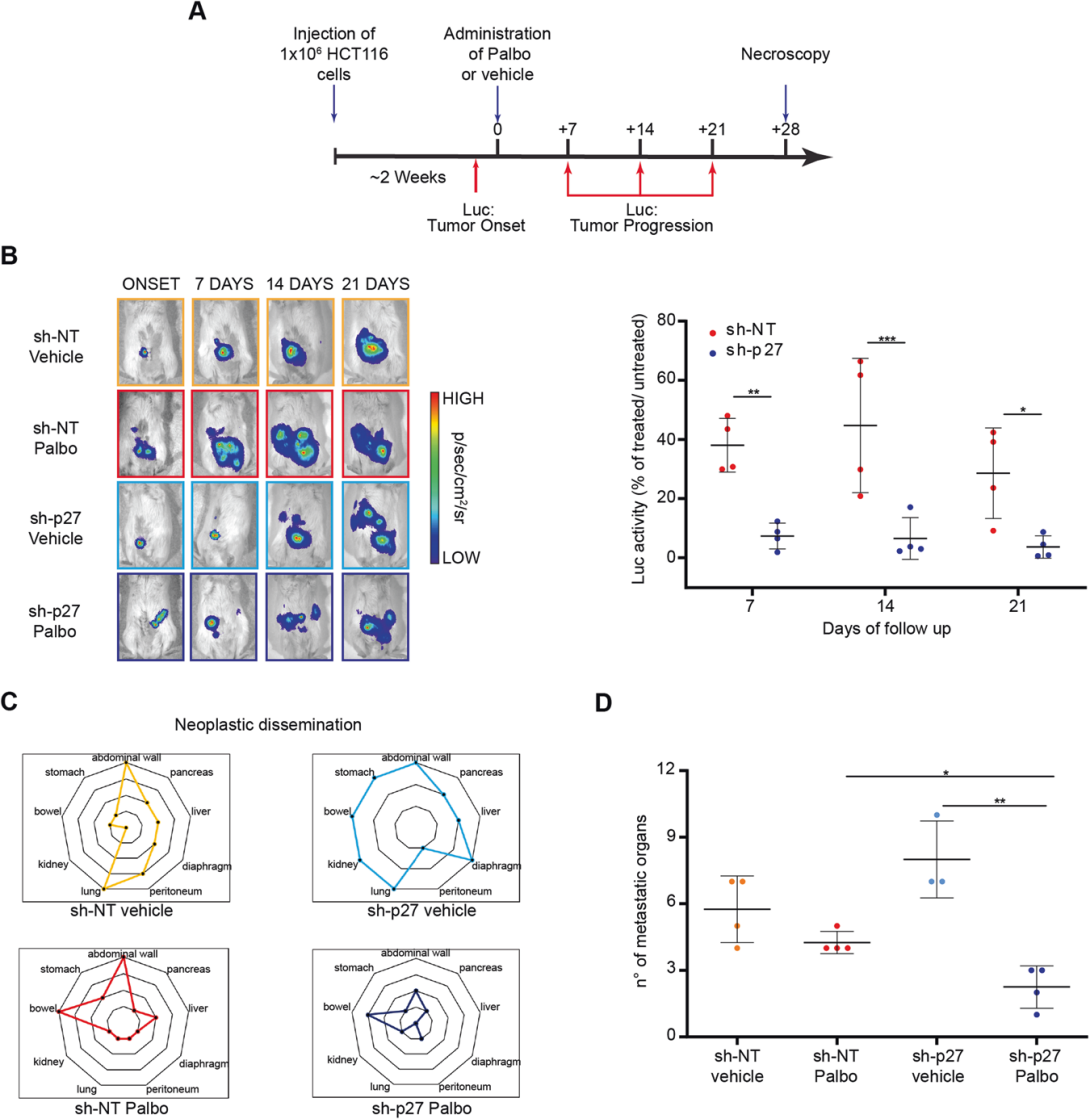
Expression of p27 modulates sensitivity to Palbo, in an orthotopic model of CRC. **A** Schematic representation of the experimental workflow used in vivo. Mice were intracecally injected with luciferase (Luc) expressing HCT-116 cells, control (sh-NT) and silenced for p27 (sh-p27). Once tumor onset was established, mice were randomly subdivided and treated 5 days/week with vehicle or Palbo for 4 weeks. Luc activity was evaluated to monitor tumor growth. **B** Left: Representative pictures of Luc activity in tumor-bearing mice at the indicated days of follow-up. Right: Dot plot reports the quantification of Luc activity at the different time points. Data are expressed as the percentage of Luc activity of Palbo treated/untreated ratio. Each dot represents a different treated mouse. **C**, **D** Radar (**C**) and dot (**D**) plots reporting the number and the distribution of metastatic organs in mice described in **A**, as assessed by histological analysis. In the figure significance was evaluated by Student’s *t*-test. Asterisks mark significant differences. *p < 0.05; **p < 0.01; ***p < 0.001.

Data so far suggested that p27 expression is modulated by and could influence the response to Palbociclib in KRAS^MUT^ CRC cells. Based on these results, we moved to an in vivo orthotopic model of CRC. Luciferase (Luc) overexpressing HCT-116 KRAS^MUT^ cells, silenced for p27 (sh-p27) or control (sh-NT) (Supplementary Fig. S4A), were implanted into the cecum wall of NSG mice and tumor growth monitored. Once tumors became detectable, mice were randomly divided in two groups, treated with vehicle or Palbo (100 mg/kg, 5 days/week for 4 weeks), and weekly monitored for Luc activity (Fig. 3A). Results showed that, as expected, p27 silencing increased tumor growth compared to control in the group of vehicle-treated animals (Fig. 3B and Supplementary Fig. S4B). However, in the Palbo-treated animals, p27-silenced tumors (sh-p27) responded much more effectively to the treatment than the control ones (sh-NT) (Fig. 3B and Supplementary Fig. S4B). Pathological analysis confirmed these results and showed that sh-p27 HCT-116 cells metastasized more, compared to control cells. Importantly, while tumor dissemination was not significantly hampered by Palbo treatment in control cells (Fig. 3C and D), Palbo significantly reduced the metastatic spread of sh-p27 HCT-116 cells (Fig. 3C and D). The application of the Cancer Staging System for orthotopic CRC tumors in mice [42], further confirmed a down-staging effect of Palbo, specifically and only in p27-silenced tumors (Supplementary Fig. S4C and D).

### Palbociclib resistant CRC cells display increased p27 expression

**Fig. 4:**
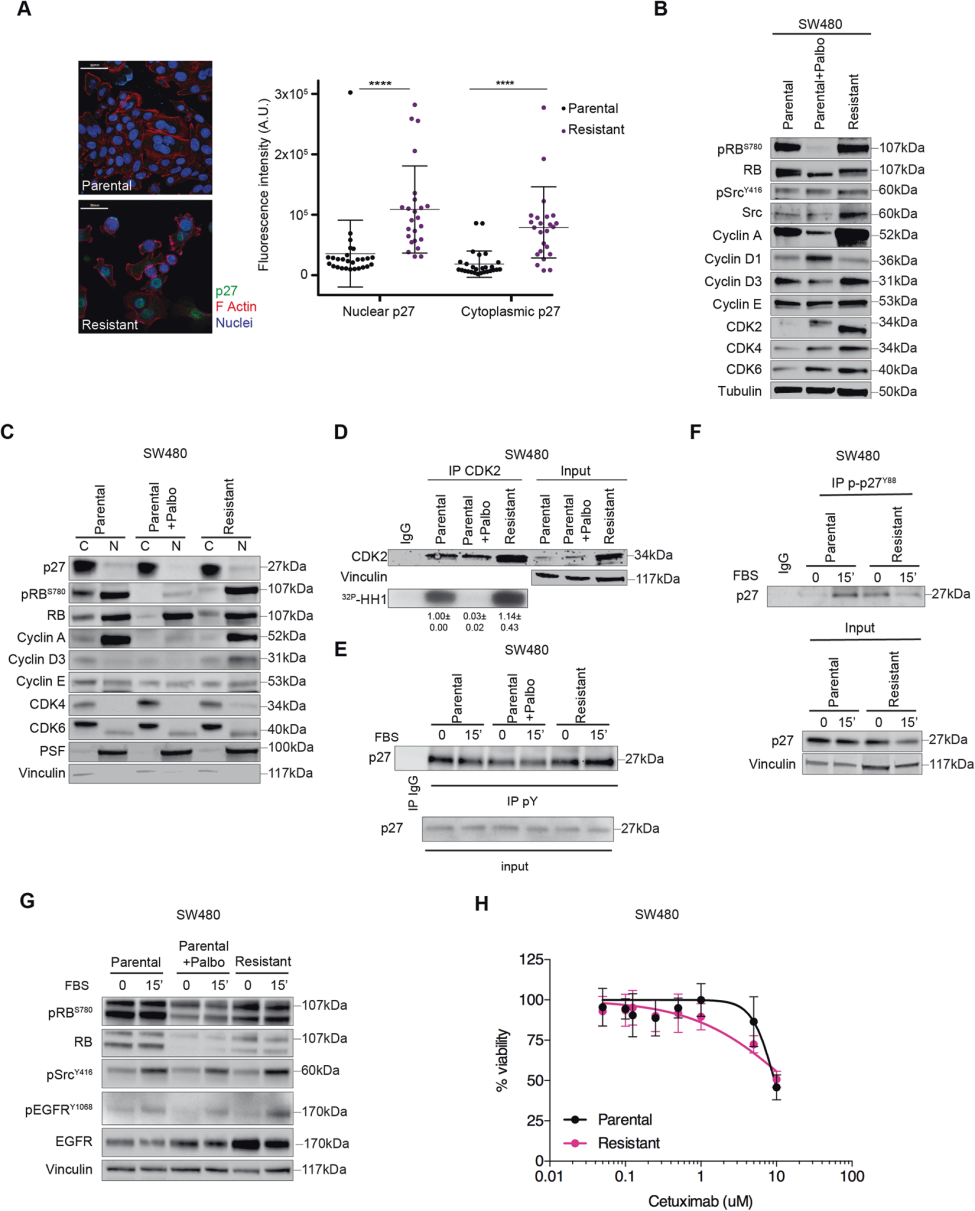
Palbo-resistant phenotype relies on phosphop27-CDK4 interaction. **A** Left: Typical images of immunofluorescence evaluating p27 expression (green) in parental (top) and resistant (bottom) SW480 cells. Staining of F-Actin (red) and nuclei (To-Pro-3, blue) was used to evaluate p27 subcellular localization. Right: Graph reports the quantification of p27 expression (expressed as arbitrary units) in nuclear and cytoplasmic compartments. Each dot corresponds to one cell. Mann–Whitney test was used for statistical analysis. **B** Western blot analysis evaluating the expression of the indicated cell-cycle proteins in SW480 cells treated or not with Palbo (2 μM, 24 h) and resistant cells cultured in presence of 2 μM Palbo. **C** Expression of the indicated proteins in the nuclear (N) and cytoplasmic (C) fractions of SW480 cells treated or not with Palbo (2 μM, 24 h) and resistant cells, as indicated. **D** Kinase assay evaluating the activity of endogenous CDK2 immunoprecipitation from parental SW480 treated or not with 2 µM Palbo for 24 h and Palbo-resistant cells cultured in the presence of 2 µM Palbo. In the kinase assay recombinant Histone H1 (^32P^-HH1) was used as substrate. Input indicates the expression of CDK2 in corresponding cell lysates and vinculin was used as loading control. Immunoglobulin G (IgG) represents the control IP using an unrelated antibody. **E** Co-immunoprecipitation (IP) analysis of endogenous thyrosin phosphorylated proteins (pY) in SW480 parental cells, treated or not with Palbo (2 μM, 24 h), and Palbo-resistant cells, serum starved (0) and released with FBS for 15’, as indicated. IP indicates the expression of pY-p27. Input stands for the expression of indicated proteins in corresponding cell lysates and vinculin was used as loading control. **F** Immunoprecipitation (IP) analysis of endogenous phosphorylated p27^Y88^ in SW480 parental and resistant cells, serum starved (0) and released with FBS for 15’. Immunoglobulin G (IgG) represents the control IP using an unrelated antibody. Input indicates the expression of total p27 in corresponding cell lysates and vinculin was used as loading control. **G** Expression of the indicated proteins and phospho-proteins in cell lysates used in the IP assays shown in **E** and **F**. **H** Graph reports the cell viability of parental (black line) and resistant (pink line) SW480 cells, treated for 72 h with increasing doses of Cetuximab and evaluated using the MTS assay. Values from untreated cells represent 100% viability. Data represent the mean (±SD) of three independent experiments performed in sextuplicate.

Data collected so far pointed to a role for p27 in the regulation of Palbo-sensitivity and also suggested that p27 could participate to the mechanisms of CDK4/6i acquired resistance. To evaluate this possibility, we generated a cellular model of Palbo-resistance, by treating SW480 cells with increasing doses of Palbo, (Supplementary Fig. S5A), as described [14]. The resistant population was continuously maintained in medium containing Palbo (2 µM) and displayed higher Palbo IC50 compared to parental cells (14.73 µM vs 10.72 µM). Resistant cells showed an increased expression of p27, both in nuclear and cytoplasmic compartments, accompanied by an increase in both CDK4 and CDK6 (Fig. 4A–C and Supplementary Fig. S5B). As observed in CDK4/6i-treated parental cells (Supplementary Fig. S3B), also Palbo resistant cells displayed unchanged CDK4 and p27 mRNA levels, compared to control cells, and only CDK6 transcript was slightly increased (Supplementary Fig. S5C). To assess whether resistance to Palbo was mediated by the observed increase in p27 and CDK4 proteins, we silenced either CDK4 or p27 and looked at their sensitivity to Palbo. Silencing of CDK4 and p27 were equally effective in reverting the resistant phenotype (Supplementary Fig. S5D). Interestingly, p27 silenced cells partially reduced CDK4 expression (Supplementary Fig. S5D), suggesting that p27 may also play a role in CDK4 protein stabilization, as reported by others [25, 26].

Exposure of parental cells to Palbo induced a strong decrease in both pRB^S780^ level and CDK2 kinase activity, not observed in Palbo resistant cells, in accord with their resistant phenotype (Fig. 4B–D).

### Targeting Src restores sensitivity to Palbociclib in KRAS^MUT^ CRC

These data suggested that p27 protein, although increased in expression, was unable to inhibit CDK2 activity, an effect that could be mediated by post-translational modification of p27. It has been reported that phosphorylation of p27 on tyrosine residues (pYp27), by Brk or Src family members, can allosterically activate CDK4-Cyclin D complexes [26, 29, 30, 43] and that Palbo cannot bind CDK4 when CDK4 is engaged in pYp27-CDK4-Cyclin D1 active complexes [32].

We thus tested if p27 was modified by tyrosine modification in Palbo resistant CRC RAS^MUT^ cells. p27 was tyrosine phosphorylated in parental CRC cells and this phosphorylation decreased under Palbo treatment, both in basal condition and after serum stimulation (Fig. 4E). Interestingly, Palbo treatment in resistant cells did not reduce pYp27-phosphorylation that, conversely, was slightly increased upon serum stimulation. Then, using an anti pY88-p27 specific antibody we confirmed that this residue was more phosphorylated in resistant cells both under basal condition and after serum stimulation (Fig. 4F).

In line with these results, using an anti pY416Src antibody that recognizes the phosphorylated active form of Src family members Src Lyn, Fyn, Lck, Yes and Hck, we observed that these proteins were equally activated by serum in parental and resistant cells, but treatment with Palbo decreased their activation only in parental cells (Fig. 4G; resistant cells are continuously maintained under Palbo). Of note, we also observed that resistant cells had higher levels of EGFR and serum activated phospho-EGFR, which could explain the higher Src activity (Fig. 4G). Given this observation, we wondered whether resistant cells could be more dependent on EGFR signaling and become, then, more sensitive than parental cells to anti-EGFR treatment. To test this hypothesis, we performed kill curves using increasing doses of the monoclonal blocking antibody Cetuximab that is currently used in clinic to treat patients. The results from these analyses indicated that Palbo-resistant cells were comparatively more sensitive than the parental ones (Fig. 4H).

Overall, collected data suggested that Src family members activation might influence the response to Palbo of CRC cells by phosphorylating p27 and participate to the establishment of Palbo-resistance. To verify this hypothesis, we used two different Src inhibitors that broadly inhibit proteins of the Src family. We selected a dose of each inhibitor that decreased Src phosphorylation without affecting cell viability and then treated cells with increasing doses of Palbo, in the presence or not of Src inhibitors (Supplementary Fig. S6A and B). Intriguingly, Src inhibition only slightly improved the efficacy of Palbo in parental cells, expressing low levels of p27, but it restored Palbo-sensitivity in resistant cells, expressing high p27 level (Supplementary Fig. S6C and D).

To confirm that the effects of Src inhibition in resistant cells were specifically due to abrogation of p27 phosphorylation, we knocked out *CDKN1*B gene in HCT-116 cells, using the CRISPR-CAS9 technology, and generated p27^KO^ HCT-116 cells. p27^KO^ HCT-116 cells were more sensitive to Palbo than parental ones and rescue experiments, re-expressing p27, significantly increased the resistance to Palbo (Supplementary Fig. S7A). Next, we tested Saracatinib efficacy in combination with Palbo (Supplementary Fig. S7B and C). Under these conditions, Saracatinib increased Palbo activity only in parental cells, in the presence of p27, while it was ineffective in p27^KO^ HCT-116 cells (Supplementary Fig. S7C).

**Fig. 5:**
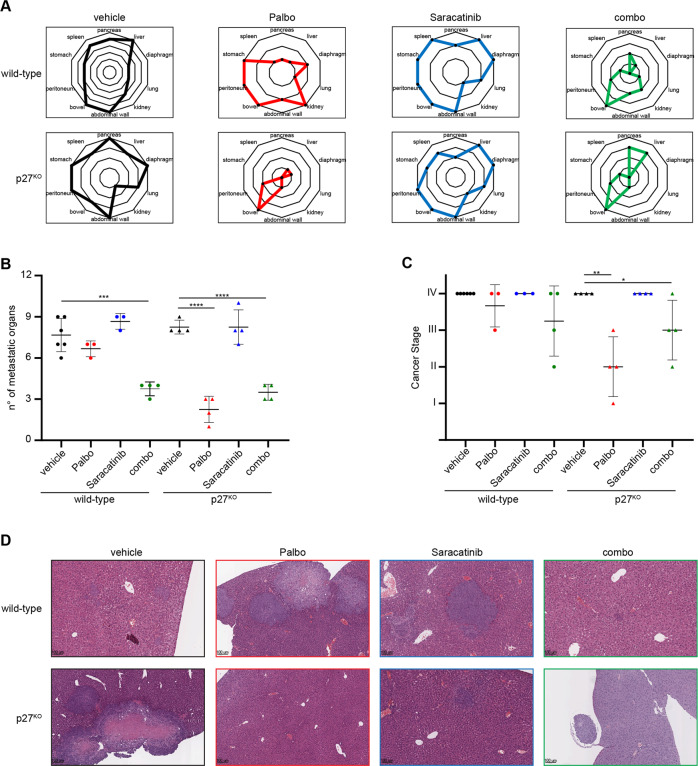
In vivo, targeting Src requires p27-expression to significantly increase CRC Palbo-sensitivity. **A**, **B** Radar (**A**) and dot (**B**) plots reporting the number and the distribution of metastatic organs in mice, as assessed by histological analysis. Mice were intracecally injected with wild-type and p27^KO^ HCT-116 cells. Once tumor onset was established, mice were randomly subdivided and treated 5 days/week with vehicle, Palbo or Saracatinib, alone or in combination (combo), as indicated. **C**, Dot plot reports tumor staging of CRC in mice evaluated according to [42], indicating the tumor spreading from the bowel (I Stage) to the peritoneum (II Stage), to mesenteric lymph node or pancreatic foci (III Stage) and the involvement of intrahepatic or lung metastasis (IV Stage). Each dot represents a different mouse from the indicated groups. **D**, Representative images of H&E analysis of liver, showing the metastatic involvement of the organ for each mice group, as indicated.

We then wondered whether the use of a combination of Palbo and Saracatinib would improve Palbo efficacy, also in vivo. We exploited again the orthotopic model of CRC, this time using parental and p27^KO^ HCT-116 cells and, once tumors appeared, divided mice in four groups: vehicle, Palbo, Saracatinib and the combo. We used a low dose of Saracatinib (25 mg/kg, twice a week), in order to see its effect in combination with Palbo. It is to mention that we observed some toxicity in Saracatinib treatment arms, which was quite unexpected since its use in patients was reported to be well tolerated [44, 45]. After 4 weeks of treatment, mice were sacrificed and tumor stage assessed. Mice implanted with p27^WT^ HCT-116 cells were quite resistant to Palbo treatment, but the combination with Saracatinib significantly reduced disease dissemination, with visible, although not significant, stage reduction (Fig. 5A–C). The analysis of livers explanted at necroscopy clearly indicated that response to Palbo was not able to reduce dissemination in mice implanted with p27^WT^ HCT-116 cells, but the addition of Saracatinib greatly improved the response, almost clearing the liver from metastatic invasion in this aggressive model of KRAS^MUT^ CRC (Fig. 5D). On the other hand, when p27^KO^ HCT-116 cells were implanted, Palbo treatment alone was highly effective and the addition of Saracatinib did not add any beneficial effect to the single treatment (Fig. 5A–D), confirming the results obtained in vitro.

**Fig. 6:**
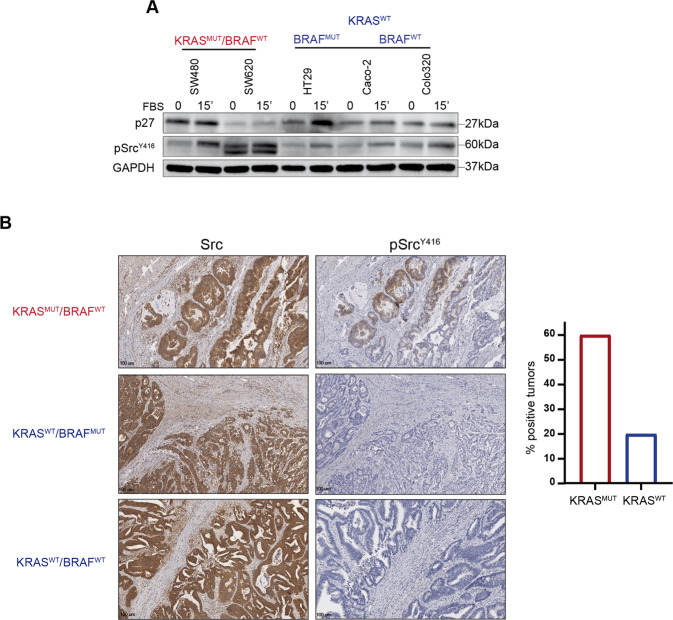
RAS^MUT^ CRC samples display increased Src activation. **A** Western Blot analysis evaluating p27 and phosphorylated Src (pSrc^Y416^) expression in CRC cell lines harboring *KRAS* mutation (SW480, SW620) or not (HT29, Caco-2, Colo320), as indicated. Cells were collected after serum starvation (0) and release with FBS for 15’, as indicated. GAPDH was used as loading control. **B** Left: Representative images of Src and pSrc^Y416^ expression evaluated by IHC in CRC samples harboring or not mutation of *KRAS* and *BRAF*, as indicated. Right: Graph reports the percentage of CRC samples showing activation of Src (pSrc^Y416^) compared to the mutational status of *RAS*.

So far, our data suggest that p27 expression plays a pivotal role in regulating the response to Palbo in KRAS^MUT^, but not in KRAS^WT^ and BRAF^MUT^ CRC cells and that this function is mediated by Src-dependent tyrosine phosphorylation. Accordingly, we observed that *KRAS* mutated cell lines were the ones expressing higher and more activated levels of Src (Fig. 6A). Moreover, among tumor samples immunostained for activated Src (*n* = 35), KRAS^MUT^ CRC displayed higher levels of active Src, compared to KRAS^WT^, either BRAF^MUT^ or BRAF^WT^ (Fig. 6B). Of note, none of the seven BRAF^MUT^ analyzed samples scored positive at all for activated Src expression.

Overall, these data support a model in which Src-dependent pYp27 phosphorylation regulates the response and drives the resistance to Palbo, in KRAS^MUT^ CRC cells.

## Discussion

Here, we investigated the mechanism linking p27 expression and Palbo-response in CRC cells and showed that high p27 expression decreases the activity of Palbo in KRAS^MUT^ cells, both in vitro and in vivo. Many observations in literature have shown that active cyclin D/CDK4 tolerate the presence of p27 within the complex, leading to the idea that cyclin D/CDK4 may titrate inhibitory p27 away from CDK2, which, in turn, was then free to phosphorylate its substrates and, eventually, drive cell cycle progression [25, 46]. However, recent data also showed that p27 is not merely a non-inhibitor for cyclin D/CDK4, but it allosterically activates the complex remodeling the catalytic ATP binding site of CDK4, eventually increasing the ATP processing. This effect, specific to CDK4 and not to CDK2, was reported to be primarily induced by tyrosine phosphorylation of p27, either on Tyr 74 or 88 [32]. Importantly, crystal structure experiments showed that, beyond decreasing its ability to inhibit the cyclin/CDKs complexes, pY-p27 precluded Palbo from inhibiting cyclin D/CDK4 complexes [32, 43] and that Palbo mostly acts as an inhibitor of the CDK4 monomer [32]. Overall, our data are in line with these observations and support the possibility that pYp27 phosphorylation is a key event in the onset of acquired resistance to CDK4/6i in CRC. Blain and collaborators reported similar observation in breast cancer, where the scenario seemed to be however quite different from what we observed in CRC. Indeed, in Palbo-sensitive breast cancer, p27 levels directly correlated with Palbo-sensitivity [47]. In this model, at longer time points of Palbo-treatment, p27 levels decreased and the ratio of pY88/p27 increased, in parallel with a restart of cell proliferation. pY88-p27, although still able to bind CDK2, becomes then an easy target for T187 phosphorylation, eventually leading to p27 degradation, CDK2 activity elevation and cell proliferation. The heavily different molecular armamentarium of these two cancer types, particularly the different drivers and the main activated pathways, easily explains why p27 plays such different roles in these two tumoral settings. For instance, while *KRAS* mutation is very frequent in CRC (about 40% of the cases), it is much less frequent in breast cancer and, in particular, in ER positive breast cancers (less than 1%), the tumor subtype in which CDK4/6i are more active [47]. Similarly, while Src activation is a key feature in both early and advanced CRC [48], in breast cancer it is more specifically connected with bone metastatic dissemination [49].

An intriguing observation resulting from our work, for which we do not have a full explanation yet, is that p27 confers resistance to Palbo only in KRAS^MUT^ and not in BRAF^MUT^ cells. Since these proteins belong to the same pathway one could expect that p27 exerted similar function(s) in both contexts. However, this is not very surprising since it is well known that KRAS^MUT^ and BRAF^MUT^ identify two quite different molecular subtypes of CRC [1]. We can speculate that only KRAS^MUT^, but not BRAF^MUT^, has the ability to co-activate the Src pathway, as already demonstrated in pancreatic cancer [50]. This hypothesis will require formal demonstration, but it is in line with the data that we obtained in CRC cell lines and primary tumors (Fig. 6) and with the notion that, both in pancreatic cancer and CRC, Src activation cooperates with KRAS^MUT^, correlating with tumor dissemination and poor prognosis [51, 52]. Since the analyses we performed do not discriminate among the different Src family members, an interesting point that will certainly deserve further investigation is the study of which Src protein(s) may be specifically upregulated and involved in the processes that we observed in CRC tumors. However, our data overall suggest that activation of a Src kinase could actively contribute to disease progression in KRAS^MUT^ CRC, a possibility that will certainly need further investigation and validation in larger cohorts of human samples and patients.

The addition of CDK4/6i to endocrine therapy in breast cancer patients has led to significant improvement of progression-free survival. Based on these promising results, great hopes are now pinned on the use of CDK4/6i in different cancers types, especially when used in combination with other targeted therapies or chemotherapy [17]. Accordingly, several clinical trials are currently testing CDK4/6i in association with other targeted agents in CRC patients (*e.g*. NCT03981614, NCT02703571, NCT04165031, NCT02745769). Notably, the JUNIPER trial in KRAS^MUT^ lung cancer has showed that the CDK4/6i Abemaciclib performed better than the EGFR inhibitor Erlotinib, in term of both objective responses and progression free survival (NCT02152631).

These data support the possibility that the use of CDK4/6i might be more successful in a subset of KRAS^MUT^-driven tumors and, therefore, predictive markers of response to select patients and ways to circumvent *de novo* or acquired resistance are greatly needed. In this context, our work that demonstrates in KRAS^MUT^ CRC that low p27 expression identifies high-risk patients that could benefit from the use of CDK4/6i, while high p27 expression, coupled with tyrosine phosphorylation by Src, may lead to Palbo-resistance, could be of great help in defining the clinical treatment decision tree for these patients. Our data further indicate that Src inhibition might increase Palbo activity in high-p27 expressing tumors and prevent the appearance of p27-induced CDK4/6i-resistance. Notably, Saracatinib has shown little efficacy as a single agent in previously treated metastatic colorectal cancer patients, but it is known for its high tolerability [44, 45]. The fact that also Cetuximab, although at very high doses, is more effective on resistant cells suggest that, on one side, increased expression and activation of EGFR could be responsible for the downstream increased Src activation observed in Palbo-resistant cells and, on the other, that now this signaling axis (EGFR-Src) might represent a synthetic lethal target together with the inhibition of CDK4/6 in these cells.

Overall, our findings indicate that in the context of KRAS^MUT^ CRC Palbo-resistance could be overcome by the concomitant use of Src inhibitors and that evaluation of p27 expression in tumor tissue might be sufficient to select KRAS^MUT^ CRC patients who may benefit from administration of CDK4/6 inhibitors, alone or in combination with Src inhibitors.

## Supplementary information

Supplementary Information
