# Supplementary material for: p27kip1 expression and phosphorylation dictate Palbociclib sensitivity in KRAS-mutated colorectal cancer
Source: Cell Death Dis. 2021 Oct 15;12(10):951. doi: 10.1038/s41419-021-04241-2 (PMC8519959; doi:10.1038/s41419-021-04241-2)
Supplement: Supplementary file 1 — Supplementary Information [file 41419_2021_4241_MOESM1_ESM.pdf]

SUPPLEMENTARY INFORMATION

p27kip1 expression and phosphorylation dictate Palbociclib sensitivity in KRAS-mutated Colorectal Cancer

Gian Luca Rampioni Vinciguerra, Alessandra Dall'Acqua, Ilenia Segatto, Maria Chiara Mattevi, Francesca Russo, Andrea Favero, Roberto Cirombella, Giorgia Mungo, Davide Viotto, Javad Karimbayli, Margherita Pesce, Andrea Vecchione, Barbara Belletti, and Gustavo Baldassarre.

CONTENTS:

**Supplementary Figures 1 – 7**	**Page 2 - 8**
**Supplementary Materials and Methods**	**Page 9 - 18**
**Supplementary References**	**Page 18**

SUPPLEMENTARY FIGURE LEGENDS

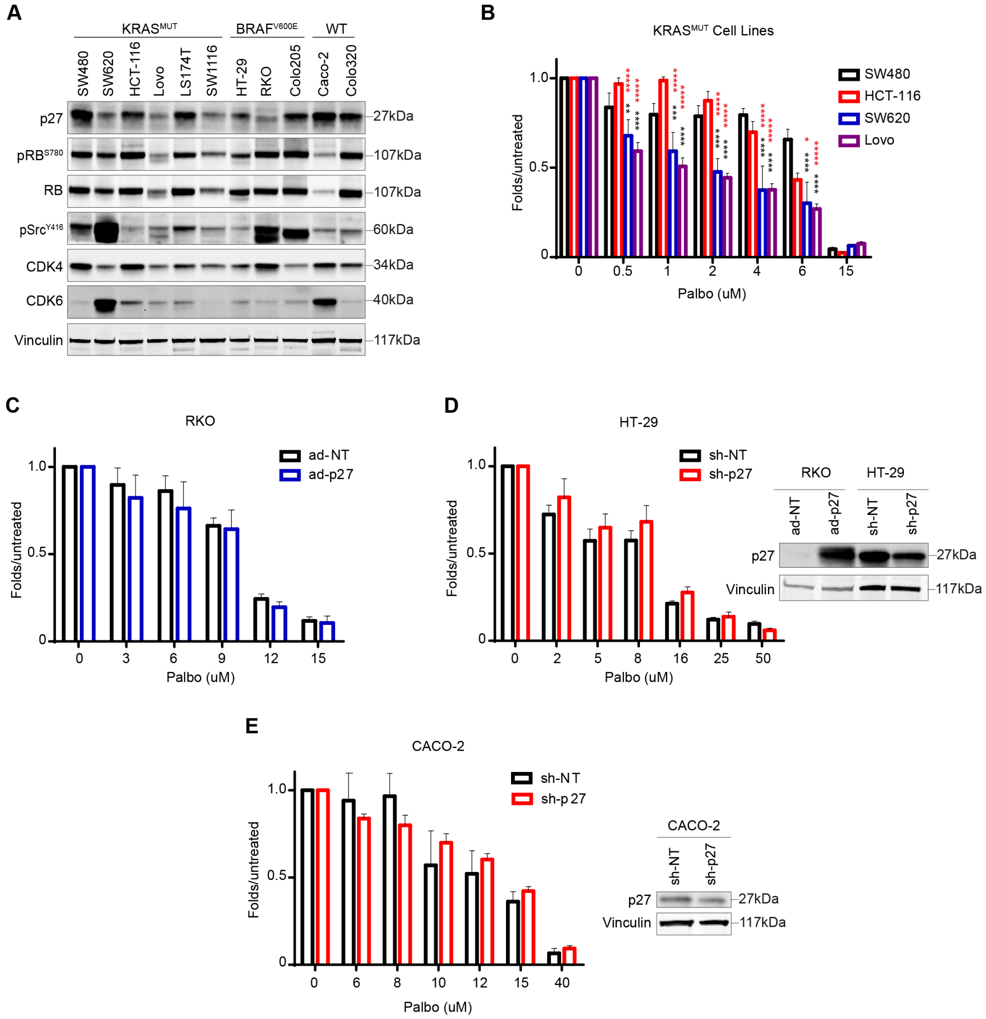

Supplementary Figure 1. p27 modulates Palbo response in KRAS^{MUT} CRC cell lines. A, Western blot analysis evaluating the expression of the indicated proteins in the indicated CRC cell lines grouped according to KRAS and BRAF mutational status. Vinculin was used as loading control. **B,** Dose-response curve evaluating Palbo sensitivity in cell lines with high (SW480, HCT-116) or low p27 expression (SW620, Lovo). Black and red asterisks indicate statistical significance referred to SW480 or HCT-116, respectively. **C**, Dose-response curve of KRAS^{WT}/BRAF^{V600E} RKO cells transduced with control (ad-NT) or p27 (ad-p27) expressing adenoviral particles, treated for 72 hours with increasing doses of Palbo, as indicated. **D**, Left: Dose-response curve of KRAS^{WT}/BRAF^{V600E} HT-29 cells control (sh-NT) and silenced for p27 (sh-p27), treated for 72 hours with increasing dose of Palbo, as indicated. Right: WB analysis of p27 in lysates from cells used for the dose-response curve of **C** and **D**. **E**, Left: Dose-response curve of KRAS^{WT}/BRAF^{WT} Caco-2 cells control (sh-

NT) and silenced for p27 (sh-p27), treated for 72 hours with increasing doses of Palbo, as indicated. Right: Western blot analysis of p27 in the same cells used for the dose-response curve. In D and E vinculin was used as loading control. In all dose-response curves, cell viability was measured by MTS assay and data show the percentage of viable treated cells compared to untreated cells. Data are the mean of at least n=3 independent experiments and Student's t-test was used to calculate statistical analysis. Asterisks indicate significant differences. * p <0.05; ** p <0.01; *** p <0.001; **** p <0.0001.

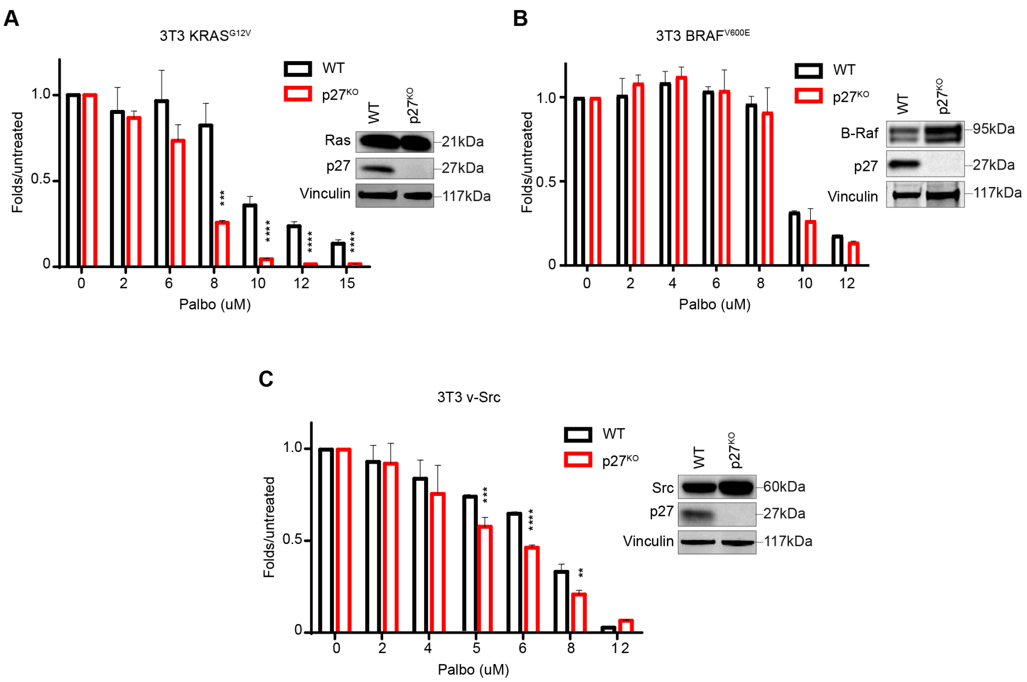

Supplementary Figure 2. p27 affects Palbo response in transformed 3T3 cells. A-C, Left: Dose-response curves of 3T3 cells WT or KO for p27 ($p27^{KO}$), stably expressing $KRAS^{G12V}$ (A), $BRAF^{V600E}$ (B) or v-Src (C), treated for 48 hours with increasing doses of Palbo, as indicated. Right: Representative Western blot (WB) analysis of p27 and each oncogene in the cells used in each dose-response curve. Vinculin was used as loading control. In all dose-response curves, cell viability was measured by MTS assay and data show the percentage of viable treated cells compared to untreated cells. Data are the mean of at least n=3 independent experiments and Student's t-test was used to calculate statistical analysis. Asterisks indicate significant differences. ** p <0.01; *** p <0.001; **** p <0.0001.

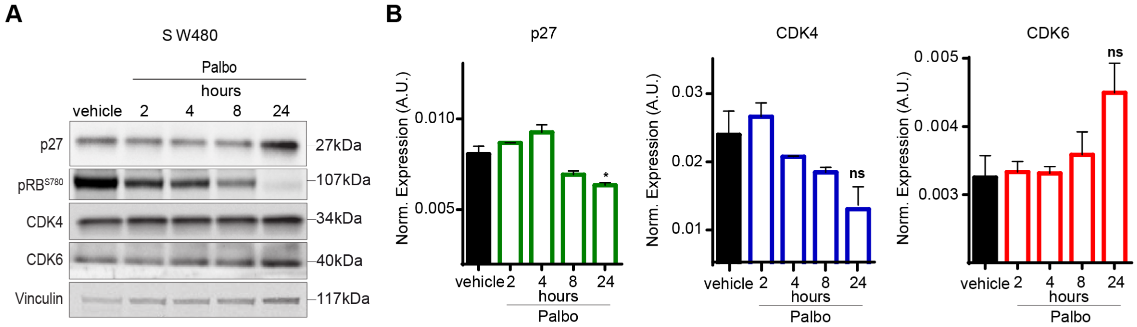

Supplementary Figure 3. Palbo treatment induces an increase of p27. A, Western Blot analysis of the indicated proteins in lysates from SW480 parental cells treated with vehicle or 2μM Palbo for the indicated times. Vinculin was used as loading control. **B,** Graphs report the normalized expression of p27, CDK4 and CDK6 mRNA evaluated by qRT-PCR analysis in SW480 parental cells treated or not with Palbo, as described in A. Data represent the normalized (to ACTB) mean value (±SD) of three different experiments performed in duplicate and are expressed as arbitrary units (AU). Statistical significance is calculated with Student's t-test and expressed as not-significant (ns) or * $p < 0.05$.

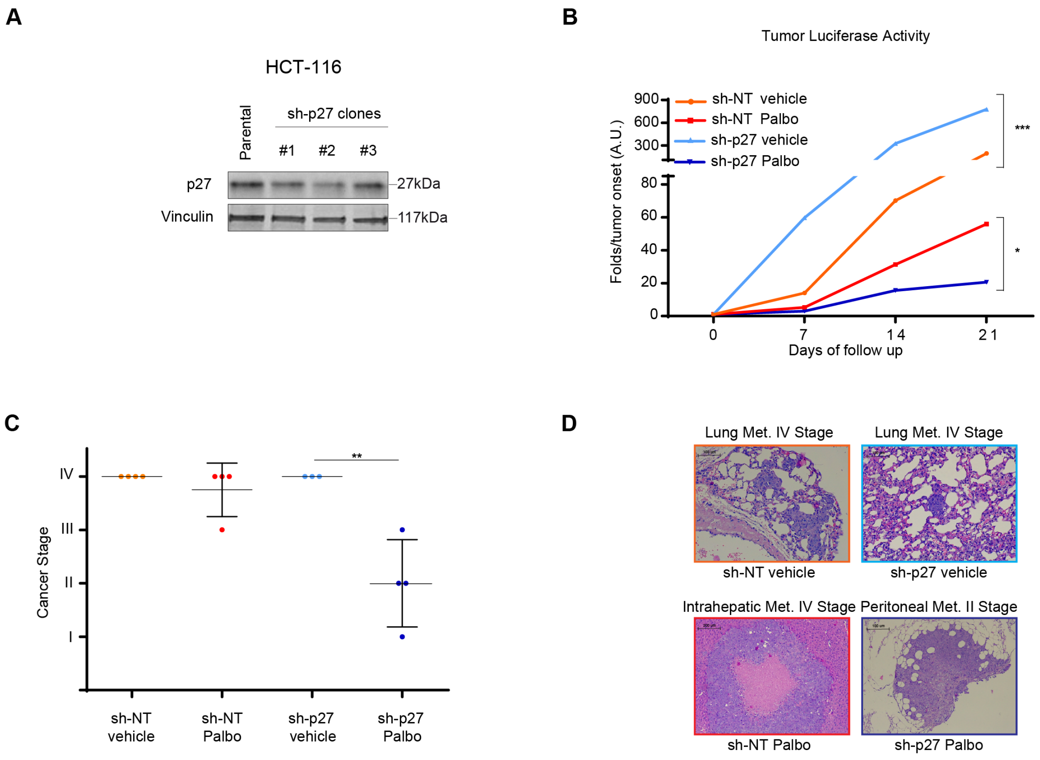

Supplementary Figure 4. ***In vivo*, Palbo treatment induces tumor downstaging in p27-silenced CRC. A,** Western blot analysis of p27 in lysates from HCT-116 parental cells and clones stably silenced for p27 (sh-p27). Clone#2 was chosen for the *in vivo* experiments. Vinculin was used as loading control. **B,** Graph reports the luciferase activity in control and p27-silenced tumors in mice treated with vehicle or Palbo at different days of follow-up as described in **Figure 3A**. Data represent the mean of Luc activity in each group (n=3 or 4) folded on the one recorded at tumor onset. **C**, Dot plot reports tumor staging of CRC in mice evaluated as reported by others[39], indicating the tumor spreading from the bowel (I Stage) to the peritoneum (II Stage), to mesenteric lymph node or pancreatic foci (III Stage) and the involvement of intrahepatic or lung metastasis (IV Stage). Each dot represents a different mouse from the indicated groups. **D**, Representative images of H&E analysis showing common findings of metastatic distant organs for each mice group as described in **C.**

In all graphs, Student's t-test was used to calculate statistical analysis and asterisks indicate significant differences. * $p < 0.05$; ** $p < 0.01$; *** $p < 0.001$.

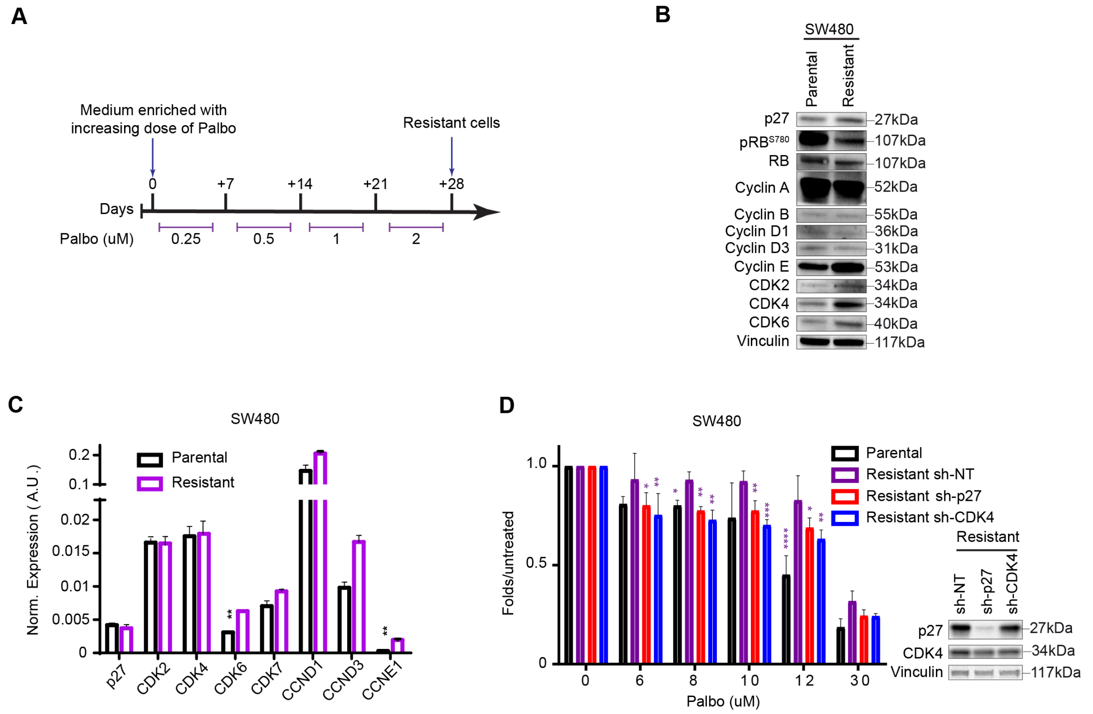

Supplementary Figure 5. p27/CDK4 interaction mediates Palbo-resistance. A, Schematic representation of the experimental workflow used to induce Palbo-resistance in SW480 cells. **B**, Western blot analysis evaluating the expression of the indicated proteins in lysates from parental and resistant SW480 cells. Vinculin was used as loading control. **C**, qRT-PCR analysis of a panel of cell cycle related genes in SW480 parental and resistant cells. Data represent the normalized (to ACTB) mean value (±SD) of three different experiments performed in duplicate and are expressed as arbitrary units (AU), statistical significance is calculated using Student's t-test (** $p<0.01$). **D**, left: Dose-response curve of SW480 parental and resistant cells transduced with control sh RNAs (sh-NT) or with shRNAs specific for p27 (sh-p27) or CDK4 (sh-CDK4) and then treated for 72 hours with increasing doses of Palbo, as indicated. Cell viability was measured by MTS assay and data are the mean of three experiment and report the percentage of viable cells compared to untreated cells. Asterisks indicate the statistical significance respect to the resistant cells, calculated using the Student's t-test. * $p <0.05$; ** $p <0.01$; *** $p <0.001$; **** $p <0.0001$. Right: Western blot analysis of indicated proteins of lysates from the same cells used for the dose-response curve. Vinculin was used as loading control.

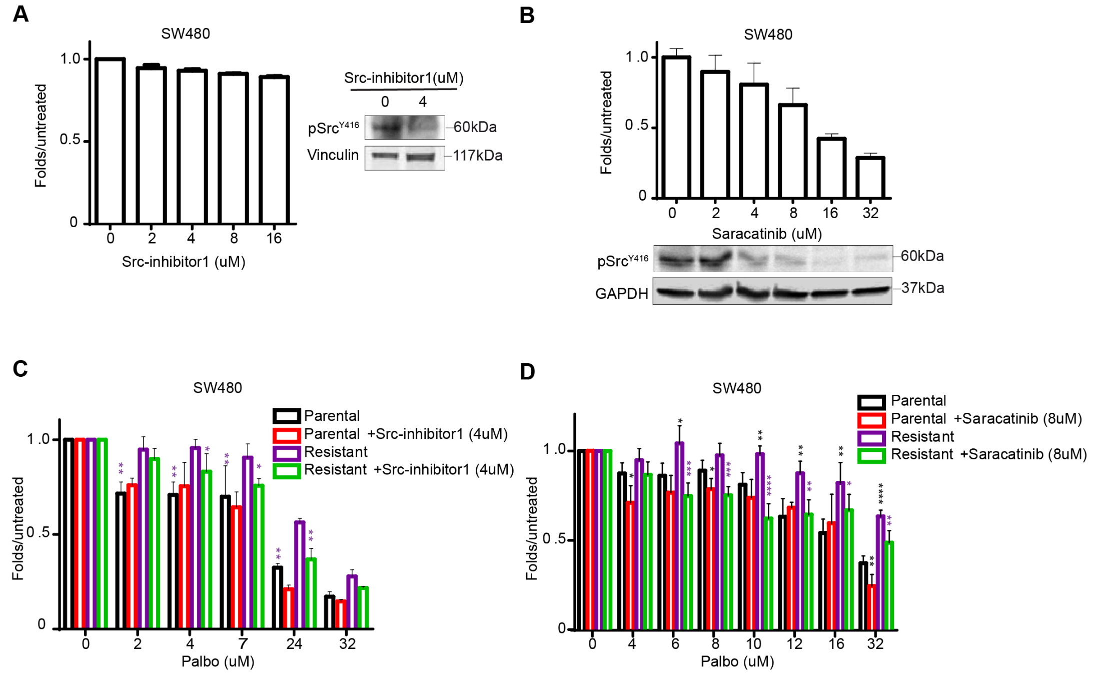

Supplementary Figure 6. Targeting Src restores Palbo-sensitivity in resistant cells. A, Left: Dose-response curve of SW480 cells treated for 72 hours with increasing doses of Src-inhibitor1, as indicated. Right: Western blot analysis evaluating of pSrc^{Y416} expression in SW480 cells untreated and treated with 4μM of Src-inhibitor1. Vinculin was used as loading control. **B,** Dose-response curve of SW480 cells treated for 72 hours with increasing doses of Saracatinib, as indicated. On the bottom**,** representative Western blot analysis evaluating of pSrc^{Y416} expression in SW480 cells lysates from the same experiment. GAPDH was used as loading control. **C,** Dose-response curve of SW480 parental and resistant cells treated for 72 hours with increasing doses of Palbo in combination or not with Src-inhibitor1 used at the fixed dose of 4μM, as indicated. **D**, Dose-response curve of parental and resistant SW480 cells treated for 72 hours with increasing doses of Palbo, in combination or not with Saracatinib 8μM, as indicated. In **C** and **D**, Black and purple asterisks indicate statistical significance referred to parental or resistant cells, respectively.

In all dose-response curves, cell viability was measured by MTS assay and data report the percentage of viable treated cells compared to untreated cells. Data are the mean of three experiments and Student's t-test was used for statistical analysis. Asterisks indicate significant differences. * $p < 0.05$; ** $p < 0.01$; *** $p < 0.001$; **** $p < 0.0001$.

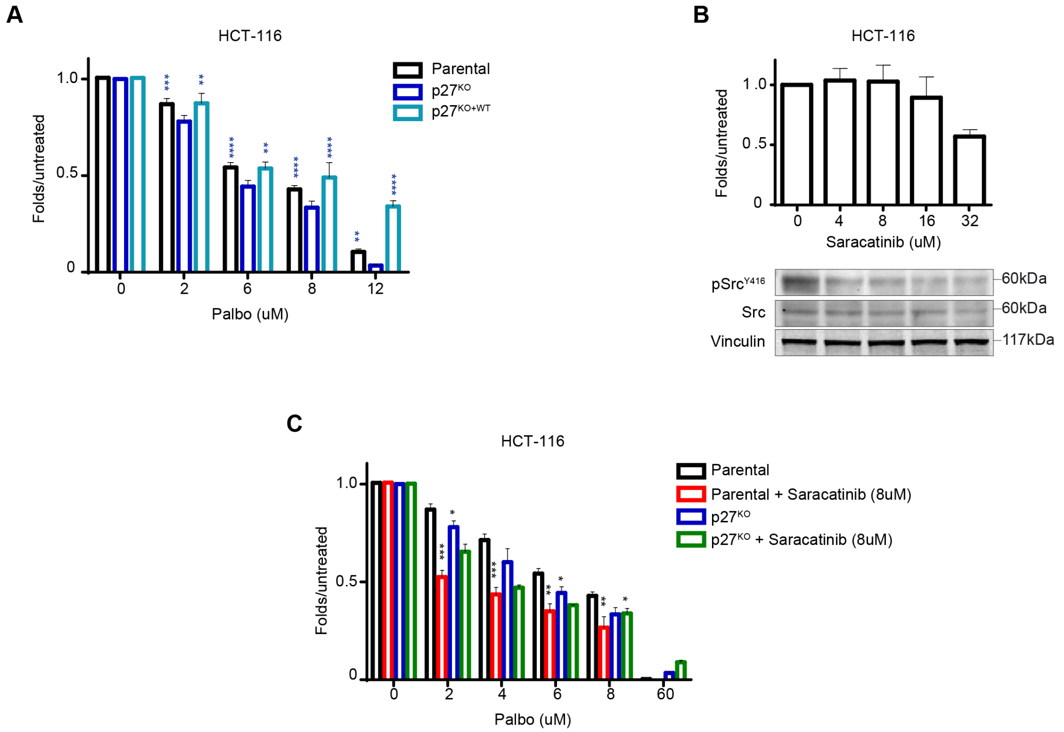

Supplementary Figure 7. Src-inhibition does not enhance Palbo-sensitivity in p27^{KO} cells. A, Dose-response curve of HCT-116 cells parental and p27^{KO} transduced with adenoviral particles expressing p27 (p27^{KO+WT}) or not (p27^{KO}), treated for 72 hours with increasing dose of Palbo, as indicated. Blue asterisks indicate the statistical significance respect to p27 ^{KO} cells. **B,** Top: Dose-response curve of HCT-116 cells treated for 72 hours with increasing dose of Saracatinib, as indicated. Bottom: Western blot analysis of Src and pSrc^{Y416} in HCT-116 cells lysates from the same experiment. Vinculin was used as loading control.

C, Dose-response curve of HCT-116 parental and p27^{KO} cells treated for 72 hours with increasing doses of Palbociclib in combination or not with Saracatinib 8μM, as indicated. Asterisks indicate the statistical significance respect to the parental cells. * $p < 0.05$; ** $p < 0.01$; *** $p < 0.001$.

MATERIALS AND METHODS

Histological analysis and Immunofluorescence

Histological sections (4 µm thick) were cut from the paraffin blocks, deparaffinized with xylene, and stained with hematoxylin and eosin, according to standard procedures. For immunohistochemical analysis, histological sections were deparaffinized and rehydrated through serial ethanol treatments. Slides were immersed in EnVision flex target retrieval solution high pH (Agilent) to enhance antigen retrieval. Endogenous peroxidase was blocked with 3% hydrogen peroxide for 15 minutes (min). Detection of p27 was performed with primary antibody clone 57 (cat. # 556049, BD Transduction Laboratories, dilution, 1:100); Src with primary antibody clone 36D10 (cat. #2109, Cell Signaling Technology, dilution 1:300); $pSrc^{Y416}$ with primary antibody clone D49G4 (cat. #6943, Cell Signaling Technology, dilution 1:300), followed by Envision FLEX/HRP (Agilent) according to manufacturer's protocol and standard procedures.

For Immunofluorescence analyses on cultured cells, cells were plated on coverslips, fixed 20 min in 4% paraformaldehyde (PFA) permeabilized with 0.2% Triton X-100 (Sigma-Aldrich) for 5 min and then incubated with primary antibodies, as reported above and previously described [36]. Briefly, incubation with primary antibodies was performed over-night (ON) at 4°C, followed by 1 hour (h) at room-temperature (RT) with secondary antibody (AlexaFluor® 488-, 546-, 568- or 633-conjugated, Invitrogen). Nuclei were counterstained using TO-PRO-3 (Invitrogen) for 30 min at RT. Staining for F-Actin (AlexaFluor® 546 Phalloidin Invitrogen) was performed 1h at RT after the incubation with secondary antibodies. Primary antibody used was: p27 (BD Transduction Laboratories # 556049). Samples were analyzed using a

confocal laser-scanning microscope (TSP8, Leica) interfaced with a Leica fluorescent microscope. Collected images were analyzed and p27 expression was quantified using the LAS (Leica) and the Volocity® (PerkinElmer) softwares.

Cell culture and generation of Palbociclib resistant cells

Caco-2, HCT116, HCT-116-Luc2, HT29, Lovo, RKO, SW480 and SW620 colorectal cancer cell lines were obtained from the American Type Culture Collection (ATCC) and were maintained in RPMI 1640 (Sigma-Aldrich) supplemented with 10% fetal bovine serum (FBS, Carlo Erba) and 1% penicillin and streptomycin (PS, Lonza).

SW480 Palbociclib resistant cells were generated by continuous treatment with increasing dose (from 0.1 to 2 μM) of Palbociclib (PD-0332991 hydrochloride, Clinisciences) for 4 weeks and isolating a resistant population that was then maintained in medium containing 2μM Palbociclib, as described [14]. Resistant phenotype was confirmed by Kinase assay in presence of Palbociclib as described above.

3T3 WT or $p27^{KO}$ fibroblasts were obtained from primary mouse embryonic fibroblasts (MEF) following up the 3T3 immortalization protocol, as previously described [34,35] and cultured in DMEM (Sigma-Aldrich) supplemented with 10% FBS (Carlo Erba) and 1% PS (Lonza). 293-FT cells were obtained from Invitrogen (#R70007, Thermo Fisher Scientific), and maintained in DMEM high glucose (Sigma-Aldrich), supplemented with 10% FBS (Carlo Erba), 2 mM Non-Essential Amino Acids (NEAA, Sigma-Aldrich), 2 mM L-glutamine (Sigma-Aldrich), 2 mM Sodium Pyruvate, and 500 μg/ml G418 (Sigma-Aldrich). Cell lines were grown at

37 °C, under 5% CO2, in humidified incubators and routinely tested using MycoAlert detection Kit (Lonza) for mycoplasma contamination. Only mycoplasma negative cells were used for experiments. Human cell lines were authenticated by STR analysis in 2018, according to PowerPlex® 16 HS System (Promega) protocol and using GeneMapperTM software 5 (ThermoFisher) to identify DNA STR profiles.

Transfection and generation of stable clones

293FT cells (Invitrogen, Thermo Fisher Scientific) were used for lentiviral production and grown in DMEM supplemented with 10% FBS (Carlo Erba). p27 and CDK4 silenced cells were generated by lentiviral system. pLKO vectors encoding for human shRNAs of the MISSiON system (pLKO lentiviral vector, Sigma-Aldrich) were used as described elsewhere [14]. Briefly, 293FT cells were transfected with Gag-Pol and VSV-G (Invitrogen recombinant lentivirus producing system) plus pLKO shRNA (p27: TRCN0000039930 and TRCN0000356318; CDK4: TRCN0000196698 TRCN0000010520) by standard calcium phosphate transfection protocol (Promega). After 48 and 72 hrs, conditional medium containing lentiviral particles was harvested and used to transduce target cells. HCT-116-Luc sh-p27 clones were selected in complete medium supplemented with 1.5µg/ml puromycin.

3T3 fibroblasts transformed with K-Ras^{G12V}, pM-vSrc were described previously [34,35], BRAF^{V600E} transformed cells were generated using the pBabe-BRAFV600E plasmid obtained by Dr. W Han through Addgene and selected in puromycin for one week. Selected clones were analysed for BRAF^{V600E} expression and used in dose

response curve. Recombinant adenoviruses for p27 overexpression ($p27^{WT}$, $p27^{CK-}$ and $p27^{1-170}$) were produced with the Adeno-X ViraTrak ZsGreen1 Expression System 2 (Clontech), according provider's instructions. Adenovirus production, titration and characterization were performed as previously described [50].

Generation of stable $p27^{KO}$ cell clones

To generate HCT-116 p27 KO clones, CRISPR/Cas-9 technology was used essentially as described [51]. Briefly, First, using Cas-9 lentiviral particles (LV-Ef1aCas9Blst vector, Sigma-Aldrich), stable pool of HCT-116 Cas-9 was produced. Clones were selected in complete medium supplemented with Blasticidin 8 μM. Stable HCT116 Cas-9 p27 KO pool was obtained by transduction with guide RNA (gRNA) lentiviral particles (LV04U6-gRNA:hPGK-puro-2A-tBFP, Sigma-Aldrich). The gRNA designed using Sigma-Aldrich tool to target CDKN1B was GTCCCGGGTTAACTCTTCG. Clones were selected after 72 hrs from transduction in complete medium supplemented with 1.5 μg/ml of puromycin. In order to analyze the Cas9 activity pool, DNA was isolated with Maxwell® 16 Mouse Tail DNA kit in Maxwell® 16 Instrument (Promega) to performed Next Generation Sequencing (NGS) analysis (MySeq, Illumina). To obtained p27 KO HCT-116 clones single-cell was seeded into 96-well plates. Selected, clones were analyzed by NGS and the clones resulted KO were next confirmed by Western Blot analysis of p27 expression.

Cell viability and kill curve

For dose-response curve, colorectal cancer cells were transduced with lenti- or adenoviral particles to obtain p27 silencing or overexpression and after 24 hours (hrs) seeded in 96- well culture plates at concentration of $3x10^3$ cells/well. Transformed 3T3 WT or $p27^{KO}$ cells were seeded at concentration of $1x10^3$-$3x10^3$, depending on cell type.

After 24 hrs, cells were treated with increasing doses of Palbociclib (PD-0332991 hydrochloride Clinisciences), Saracatinib (AZD0530, SelleckChem) and SRC-inhibitor-1 (S2075, Sigma-Aldrich) alone or in combination, as indicated.

Cell viability was determined at the end of treatment (48 or 72 hrs depending on the experiment, as indicated) using the MTS assay (CellTiter 96 AQueous One Solution Cell Proliferation-MTS-Assay, Promega) following manufacturer's instructions.

Preparation of protein lysates and Western blot analysis

Protein lysates and Western Blot were performed essentially as previously described [34-36]. Proteins were separated in 4-20% SDS-PAGE (Criterion TGX Precast Gel, Bio-Rad) and transferred to nitrocellulose membranes (GE Healthcare). Membranes were blocked with 5% non-fat dried milk (NFDM) in TBS-0.1% Tween-20 or in Odyssey Blocking Buffer (LI-COR, Biosciences) and incubated at 4°C overnight (ON) with primary antibodies. Primary antibodies used were: p27 (BD Transduction Laboratories #556049), p27 C-19 (Santa Cruz Biotechnology, #sc-528), CDK2 (Santa Cruz Biotechnology, #sc-6248), CDK4 (Santa Cruz Biotechnology, #sc-260), CDK6 (Santa Cruz Biotechnology, #sc-53638), Cyclin A B8 (Santa Cruz Biotechnology, #sc-271682), Cyclin B1 GNS1 (Santa Cruz Biotechnology, #sc-245) Cyclin-D1 (Calbiochem, #CC12), Cyclin-D3 (Cell Signaling Technology, #2936),

Cyclin-E1 (Santa Cruz Biotechnology, #sc-481), pY (BD Transduction Laboratories, #610000), pY1068 EGFR (Cell Signaling Technology, #3777), EGFR (Santa Cruz Biotechnology, #sc-03), pY416 SRC (Cell Signaling Technology, #2101), SRC (Santa Cruz Biotechnology, #sc-19), pS780 Rb (Santa Cruz Biotechnology, #sc-12901), panRAS (Calbiochem, #OP40), B-RAF (Santa Cruz Biotechnology, #sc-5284), PSF (Santa Cruz Biotechnology, #sc-101137), tubulin (Sigma-Aldrich, #T5168), Vinculin (Santa Cruz Biotechnology, #sc-73614), GAPDH (Cell Signaling Technology, #5174). p-p27^{Y88} was a kind gift from Ludger Hengst (Medizinische Universitat Innsbruck, Innsbruck, Austria).

Following incubation with primary antibody, membranes were washed in TBS-0.1% Tween 20 and incubated 1 hour (h) at room temperature (RT) with secondary antibodies IRconjugated (Alexa Fluor 680, Invitrogen; IRDye 800, Rockland) for infrared detection (Odyssey Infrared Detection System, LI-COR) or with the horseradish peroxidaseconjugated secondary antibodies (GE Healthcare) for ECL detection (Clarity Western ECL Substrate, Bio-Rad). The Re-Blot Plus Strong Solution (Millipore) was used to strip the membranes, when re-blotting was needed.

Quantitative Real-Time PCR (qRT-PCR)

RNA was extracted using TRIzol reagent (Invitrogen, Thermo Fisher Scientific) following manufacturer's instructions. Total RNA was quantified using the QuantiFluor RNA System (Promega). RNA was retro-transcribed (RT) using the GoScript Reverse Transcriptase (Promega) and RT reactions were run in an Opticon qRT-PCR Thermocycler (Bio-Rad). qRT–PCR analyses were performed essentially as described [36]. cDNAs were amplified using SYBR green dye-containing reaction

buffer SSoFast (Bio-Rad) and the CFX384 Touch Real-time PCR Detection System (Bio-Rad). Normalization of the data was performed using two different housekeeping genes. The following primers (Sigma-Aldrich) were used:

CDKN1B: **FW** AGATGTCAAACGTGCGAGTG; **REV** TCTCTGCAGTGCTTCTCCAA.
CDK2: **FW** CCTCCTGGGCTGCAAATA; **REV** CAGAATCTCCAGGGAATAGGG.
CDK4: **FW** GTGCAGTCGGTGGTACCTG; **REV** TTCGCTTGTGTGGGTTAAAA.
CDK6: **FW** TGATCAACTAGGAAAAATCTTGGAC; **REV** GGCAACATCTCTAGGCCAGT
CDK7: **FW** CCATGTGCTCGAATTACGG; **REV** CTTGGCAGCTGACATCCAG.
CCND1: **FW** AGAAGGAGGTCCTGCCGTCC; **REV** GGTCCAGGTAGTTCATGGCC.
CCND3: **FW** GGAAGATGCTGGCTTACTGG; **REV** AGACAGGTAGCGATCCAGGT.
CCNE1: **FW** ACAGCTTGGATTTGCTGGAC; **REV** TCTTTGGTGGAGAAGGATGG.
ACTB: **FW** CCAGAGGCGTACAGGGATAG; **REV** CCAACCGCGAGAAGATGA.
Relative expression was calculated using the comparative Ct method.

Immunoprecipitation and Kinase assay

Immunoprecipitation (IP) experiments were performed using 0.7 mg of total lysate with the primary antibody in HNTG buffer (20 mM HEPES, 150 mM NaCl, 10% Glycerol, 0.1% Triton X-100) supplemented with 1 mM Na3VO4, 10 mM NaF (Sigma-Aldrich), 1 mM DTT (Sigma-Aldrich), gently rocking ON at 4°C. Protein A or Protein G Sepharose™ 4 Fast Flow (GE Healthcare), was added during the last 2 hrs of incubation. For p-p27^{Y88}, antibody was previously mixed to Protein G Sepharose™, gently rocking ON at 4°C, to allow the conjugation and then added to total lysates as described. IPs were then washed six times in HNTG buffer and resuspended in 3X Laemmli sample buffer (5X Laemmli buffer composition: 50 mM TrisHCl pH 6.8, 2% SDS, 10% glycerol, 0.05% bromophenol blue and 125 mM

beta-mercaptoethanol). Immunoblot analysis was performed as described above. For kinase assay, cell lysates were immunoprecipitated using anti-CDK2 or control antibody, as described above. After 7 washes in HNTG buffer, two tenth of the IP was resuspended in kinase buffer solution (20 mM TrisHCl pH 6.8, 10 mM $MgCl_2$). Then, a kinase reaction solution containing the sample plus 50 μM ATP, 1μCi of γ-P^{32} ATP and specific substrate (1 μg of H1-Histone for CDK2) in kinase buffer solution was prepared. The reaction was carried out at 30°C for 30 min and then 5X Laemmli sample buffer and 50 mM of DTT was added. After denaturation at 95 °C for 10 min, proteins were loaded on a 4–20% SDS-PAGE. The gel was then dried at 80°C for 3 hours under vacuum and exposed on an autoradiographic film (GE, Amersham-Hyperfilm MP) at RT and developed after different time intervals.

Animal experimentation

Animal experimentation was reviewed and approved by the Centro di Riferimento Oncologico di Aviano (CRO) Institutional Animal Care and Use Committee (OPBA). All animal experiments were conducted in adherence to international and institutional committees' ethical guidelines. NOD.CB17-PrkdcSCID (NSG) male mice (Charles River Laboratories, 4 to 6 weeks old, n=47) were xenografted with 1×10^6 HCT-116-Luc2 (Caliper) cells either stably silenced (n=7) and not for p27 (n=8), or wild-type (n=16) and $p27^{KO}$ (n=16), as described above. Cells were resuspended in 50μl of red-phenol free RPMI and injected intra-cecum under anesthesia, as described by others [39]. To establish the tumor onset after intracecal injection, luciferase activity was measured every 3 days by intraperitoneal injection of luciferin (CycLucI, Merck #5306500001, 100mg/kg) in anesthetized mice using IVIS Spectrum In vivo imaging system by PerkinElmer.

After tumor onset, in Palbociclib experiment, sh-NT and sh-p27 injected animals were randomly divided into two groups (4 mice/group; 3 mice/group for shp27 control group) and treated with Palbociclib (PD-0332991 hydrochloride, Clinisciences, 100 mg/kg) or with vehicle (Ringer's Lactate solution) 5 days/week for 4 weeks by oral gavage.

In Palbociclib/Saracatinib experiment, wild-type and $p27^{KO}$ injected animals were randomly divided into four groups (4 mice/group; 6 mice for wild-type control group, 3 mice for wild-type Palbociclib and Saracatinib groups) and treated with vehicle (Ringer's Lactate solution) or with Palbociclib (PD-0332991 hydrochloride, Clinisciences, 100mg/kg), or with Saracatinib (Selleckchem #S1006, AZD0530, 25mg/kg) or a combination of Palbociclib and Saracatinig, 5days/week for 4 weeks by oral gavage.

Luciferase activity was measured every week to monitor tumor growth. At the time of sacrifice, all the organs collected were formalin fixed and processed for H&E staining.

Statistics and data reproducibility

Statistical significance, means, median, standard deviation were determined by using GraphPad PRISM software (version 6.01), using the most appropriate test, as specified in each figure. A minimum of three biologically independent samples was used for statistical significance. The number and type of replicates used in each experiment is specified in the figure legend. When not otherwise specified, mean and standard deviation are shown in all graphs. Significance was calculated by

Student's t-test, Fisher's exact test or Mann-Whitney two-sided test, as appropriate, and indicated by a $P<0.05$.

SUPPLEMENTARY REFERENCES

14 Haines E, Chen T, Kommajosyula N, Chen Z, Herter-Sprie GS, Cornell L et al. Palbociclib resistance confers dependence on an FGFR-MAP kinase-mTOR-driven pathway in KRAS-mutant non-small cell lung cancer. Oncotarget 2018; 9: 31572–31589.

34 Fabris L, Berton S, Pellizzari I, Segatto I, D'Andrea S, Armenia J et al. p27kip1 controls H-Ras/MAPK activation and cell cycle entry via modulation of MT stability. Proc Natl Acad Sci USA 2015; 112: 13916–13921.

35 Pellizzari I, Fabris L, Berton S, Segatto I, Citron F, D'Andrea S et al. p27kip1 expression limits H-Ras-driven transformation and tumorigenesis by both canonical and non-canonical mechanisms. Oncotarget 2016; 7: 64560–64574.

36 Dall'Acqua A, Sonego M, Pellizzari I, Pellarin I, Canzonieri V, D'Andrea S et al. CDK6 protects epithelial ovarian cancer from platinum-induced death via FOXO3 regulation. EMBO Mol Med 2017; 9: 1415–1433.

50 Schiappacassi M, Lovisa S, Lovat F, Fabris L, Colombatti A, Belletti B, *et al* (2011) Role of T198 modification in the regulation of p27(Kip1) protein stability and function. PLoS One. 6(3):e17673.

51 Sonego M, Pellarin I, Costa A, Vinciguerra GLR, Coan M, Kraut A, *et al* (2019) USP1 links platinum resistance to cancer cell dissemination by regulating Snail stability. Sci Adv 5(5):eaav3235
